# Subpathway-GMir: identifying miRNA-mediated metabolic subpathways by integrating condition-specific genes, microRNAs, and pathway topologies

**DOI:** 10.18632/oncotarget.5341

**Published:** 2015-10-12

**Authors:** Li Feng, Yanjun Xu, Yunpeng Zhang, Zeguo Sun, Junwei Han, Chunlong Zhang, Haixiu Yang, Desi Shang, Fei Su, Xinrui Shi, Shang Li, Chunquan Li, Xia Li

**Affiliations:** ^1^ College of Bioinformatics Science and Technology, Harbin Medical University, Harbin, 150081, China; ^2^ Department of Medical Informatics, Daqing Campus, Harbin Medical University, Daqing, 163319, China

**Keywords:** microRNA, mRNA, cancer, pathway identification, regulation

## Abstract

MicroRNAs (miRNAs) regulate disease-relevant metabolic pathways. However, most current pathway identification methods fail to consider miRNAs in addition to genes when analyzing pathways. We developed a powerful method called Subpathway-GMir to construct miRNA-regulated metabolic pathways and to identify miRNA-mediated subpathways by considering condition-specific genes, miRNAs, and pathway topologies. We used Subpathway-GMir to analyze two liver hepatocellular carcinomas (LIHC), one stomach adenocarcinoma (STAD), and one type 2 diabetes (T2D) data sets. Results indicate that Subpathway-GMir is more effective in identifying phenotype-associated metabolic pathways than other methods and our results are reproducible and robust. Subpathway-GMir provides a flexible platform for identifying abnormal metabolic subpathways mediated by miRNAs, and may help to clarify the roles that miRNAs play in a variety of diseases. The Subpathway-GMir method has been implemented as a freely available R package.

## INTRODUCTION

MicroRNAs (miRNAs) are short, endogenous, non-coding RNAs that regulate transcription by inhibiting the expression of target genes. MicroRNA-induced inhibition of transcription can affect the initiation, progression, and prognosis of cancers [1–5]. High-throughput technologies, such as microarray and next-generation sequencing, have helped identify many novel, disease-relevant genes and miRNAs. However, the precise mechanisms by which these biomolecules contribute to disease pathologies are often unclear. Metabolic pathway analysis is a useful approach for identifying the roles of certain biomolecules. For example, the Over-Representation Analysis method (*ORA*) [6] evaluates whether a predefined group of genes is significantly over-represented in a second gene set using such analyses as the hypergeometric test [7]. Recently, Kretschmann *et al*. conducted pathway enrichment analysis directly based on miRNAs, counting each miRNA once, regardless of the number of genes in the pathway it targets [8, 9]. Though these methods are powerful tools for pathway identification, they have limitations. The ORA and Kretschmann *et al*. method focused on only genes and miRNAs, respectively, and both neglected the joint effect exerted by genes and miRNAs on disease phenotypes. Because genes and miRNAs can work together to disrupt biological pathways and cause diseases, integrative analysis of both is necessary when identifying disease-related pathways affected by miRNAs. Databases of interactions between miRNAs and their targets, such as TarBase [10], miRecords [11], mirTarBase [12], and miR2Disease [13], are valuable resources for exploring the regulation of genes and miRNAs in disease.

Gene expression can be regulated not only by miRNAs, but also by neighboring genes. Therefore, in addition to miRNAs, pathway topology should be considered in metabolic pathway identification. Li *et al*. developed a method for incorporating pathway topologies along with miRNAs in disease mechanism models by combining sample matched miRNA-mRNA profiles with pathway structure [14]. Moreover, Li *et al*. confirmed that key local subregions, rather than entire pathways, were disordered under disease phenotypes [15, 16]. This indicates that focusing on subpathways rather than complete metabolic pathways might be more effective when identifying disease-relevant pathway topologies. Calura *et al.* developed the micrographite method to identify subpathways based on sample matched miRNA-mRNA profiles and the topology structures of pathways with linked miRNAs [17]. However, they focused on signaling rather than metabolic subpathways.

Here, we developed a new method called Subpathway-GMir. The goal of this method is to identify miRNA-mediated metabolic subpathways important in different diseases. We first analyzed the LIHC data set to evaluate the better effectiveness of Subpathway-GMir than other methods. We second analyzed the STAD data set to examine the roles of miRNAs, both individually and in clusters, in mediating crosstalking genes from multiple subpathways. We then analyzed the T2D data set to demonstrate that Subpathway-GMir is useful for diseases other than cancers. Finally, we tested the reproducibility and robustness of pathways identified by Subpathway-GMir. Subpathway-GMir has been implemented as a freely available R package at http://cran.r-project.org/web/packages/SubpathwayGMir/, and it can currently support six species for the identification of metabolic subpathways.

## RESULTS

### Reconstructing metabolic pathways embedded by miRNAs

**Figure 1:**
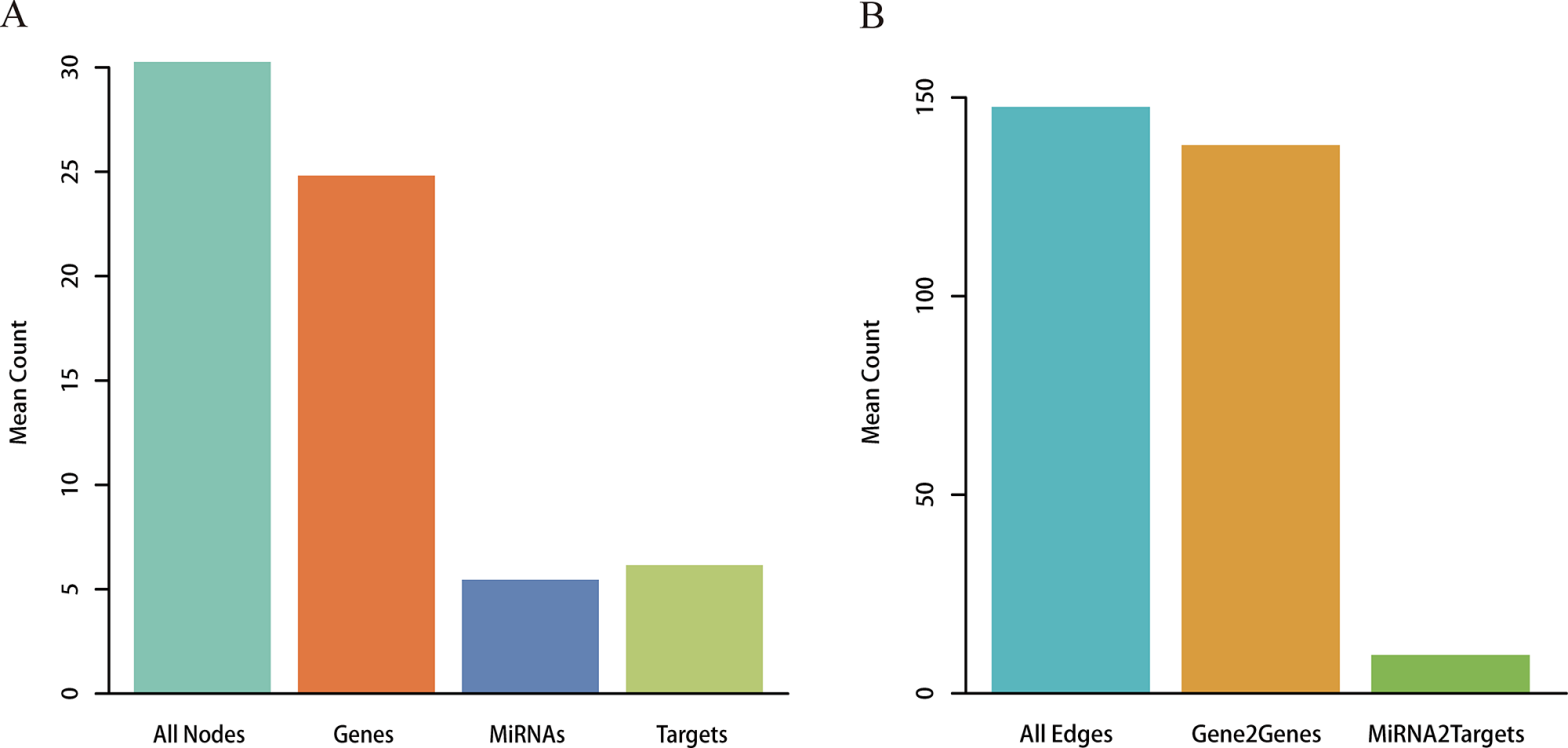
Analysis for RMPGs **A.** Node analysis. The number of all nodes, genes, miRNAs and targets within RMPGs. **B.** Edge analysis. The number of all edges (gene to gene and miRNA to target gene) within RMPGs.

Metabolic pathways were reconstructed from the Kyoto Encyclopedia of Genes and Genomes (KEGG) by integrating miRNA-target interactions verified by low-throughput experiments. A total of 101 miRNAs were embedded with the converted pathway graphs, and the final reconstructed metabolic pathway graphs (RMPGs) contained gene and miRNA nodes, gene-gene edges, and miRNA-gene edges. The “Glycolysis/Gluconeogenesis” metabolic pathway graph is shown before and after reconstruction as an example in Supplementary Figure S1. We then analyzed the average number of nodes and edges in all RMPGs. As shown in Figure 1, each RMPG contained an average of 5.45 miRNAs and 6.15 targets by node level and 9.66 miRNA-target and 137.99 gene-gene pairs by edge level.

### Identifying metabolic subpathways mediated by miRNAs in LIHC

**Figure 2:**
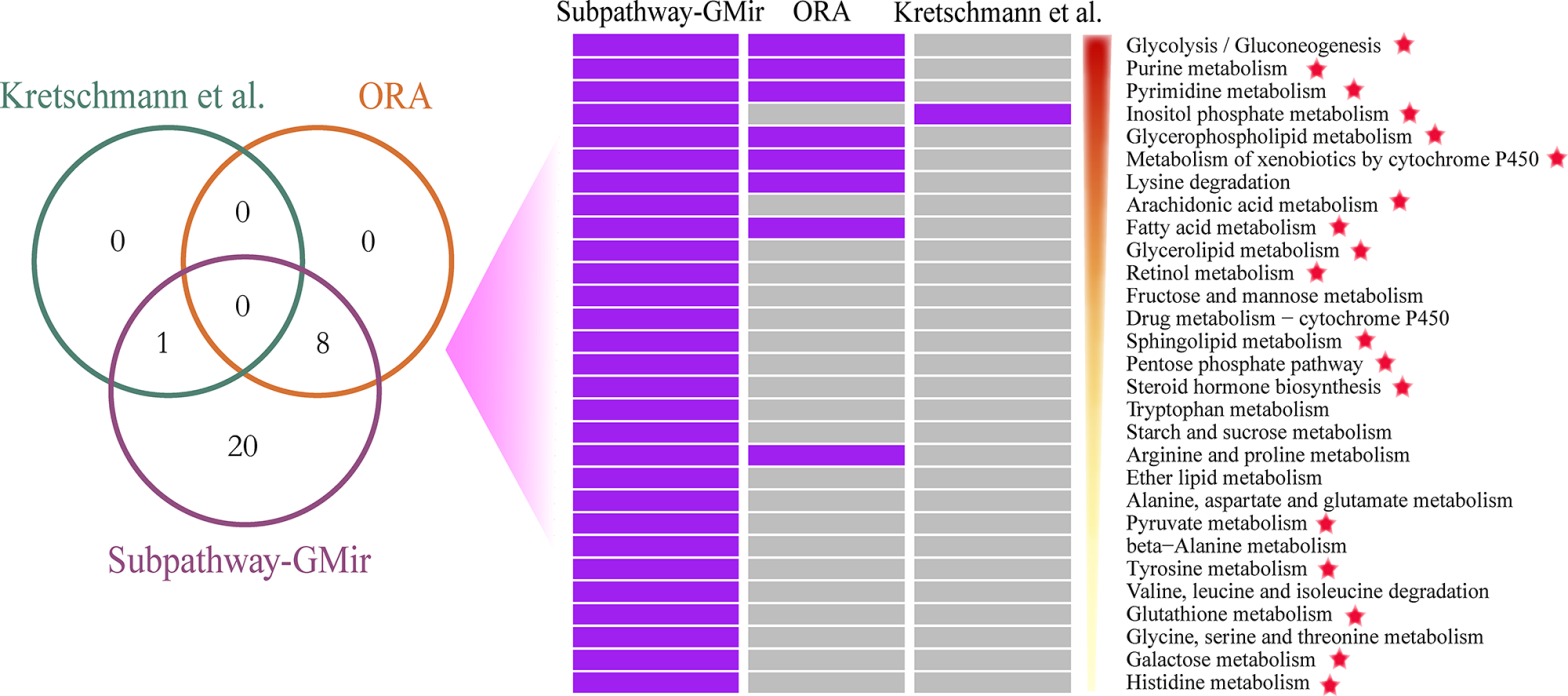
Comparison of pathways identified by Subpathway-GMir, ORA and the Kretschmann *et al*. method in LIHC data set 1 (*FDR* < 0.01) Venn diagram depicting the overlap of pathways. These pathways were sorted by statistical significance (using Subpathway-GMir) in descending order. Each row represents a pathway and each column represents a method. The purple and grey grid represents that the pathway was or wasn't identified by the method, respectively. Pathways supported by scientific studies are marked with red stars.

We first assessed the effectiveness of Subpathway-GMir. We used Subpathway-GMir to identify dysregulated subpathways involving 3357 differential genes and 394 differential miRNAs from LIHC data set 1. We identified 29 subpathways, associated with 29 distinct complete pathways at *FDR* < 0.01 (Supplementary Table S1). After manually curating from literatures, we found that 18 complete pathways are known to be associated with cancers. We then compared Subpathway-GMir results to results obtained from ORA [6] and the method used by Kretschmann *et al.* [8, 9]. The ORA and Kretschmann *et al*. method were implemented by hypergeometric test based on only genes and miRNAs, respectively, and both of them were all complete pathway identification methods. A total of 9 pathways were identified by ORA and the Kretschmann *et al*. method (*FDR* < 0.01), all of which were included in the list generated using Subpathway-GMir (Figure 2). Furthermore, 11 of the 20 LIHC pathways not detected by ORA or the Kretschmann *et al*. method are associated with cancers, and 10 of those involve cancer-related miRNAs [18]. It is obvious that Subpathway-GMir is more effective than other methods to identify LIHC-relevant metabolic pathways.

**Figure 3:**
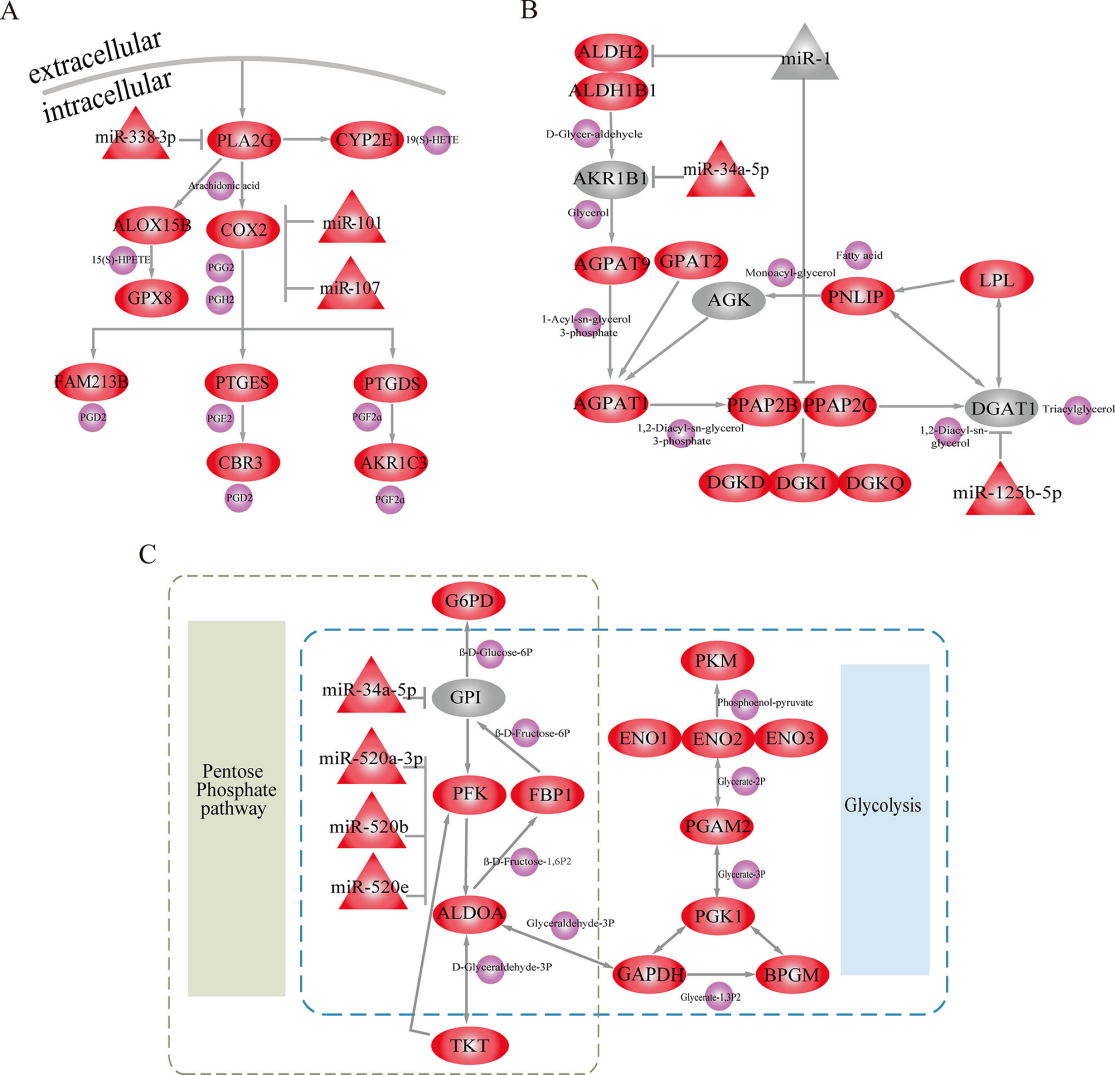
Subpathways identified using the Subpathway-GMir method Ellipse, triangle and circle nodes represent genes, miRNAs and metabolites, respectively. Red and grey nodes represent differential and non-differential genes and/or miRNAs, respectively, and purple nodes represent metabolites. **A.** Arachidonic acid metabolic subpathway (path:00590_1, *FDR* = 4.07E-12). **B.** Glycerolipid metabolic subpathway (path:00561_1, *FDR* = 1.52E-10). **C.** Pentose phosphate metabolic subpathway (path:00030_1, *FDR* = 6.28E-09).

In further analyses, we focused on three of these metabolic subpathways mediated by miRNAs. The first was the most significant subpathway, path:00590_1 (*FDR* = 4.07E-12), an important subregion within the arachidonic acid metabolic (AAM) pathway (Figure 3A). When analyzing the complete AAM pathway, neither ORA (*FDR* = 0.016) nor the Kretschmann *et al.* (*FDR* = 1) method returned a significant result. Studies demonstrate that the inhibition of AAM pathway can suppress the development and apoptosis of hepatomas [19, 20]. As the pivotal subregion of AAM pathway, path:00590_1 contained 13 consecutive differential molecules, including 10 genes and 3 miRNAs. Among these differentials were Cyclooxygenase-2 (COX-2), its downstream metabolite Prostaglandin H2 (PGH2) and miR-101. Overexpression of the differential gene (COX-2), which was at the center of path:00590_1, promotes LIHC cell growth of *in vitro* and in animal models [21]. Moreover, COX-2 catalyzes the conversion of arachidonic acid to PGH2. PGH2 synthesis facilitates the development and progression of neoplasms by inhibiting tumor angiogenesis and immune function [22–24]. The miRNA miR-101 was also an important regulator in path:00590_1, and miR-101 downregulation can promote apoptosis and suppress tumorigenicity in LIHC [25]. Normally, miR-101 inhibits COX-2 expression to promote progression of multiple cancers [26, 27]. The presence of miR-101 and COX-2 at core positions within path:00590_1 suggests that Subpathway-GMir can identify novel LIHC-relevant subpathways within larger metabolic pathways.

The second significant subpathway, path:00561_1, is part of the glycerolipid metabolic pathway (Figure 3B). Path:00561_1 was identified by Subpathway-GMir at *FDR* = 1.52E-10, but the glycerolipid metabolic pathway as a whole was detected by neither ORA (*FDR* = 0.015) nor the Kretschmann *et al*. (*FDR* = 1) method. Path:00561_1 contained miR-1, a non-differential miRNA. It also contained the initial position gene Aldehyde Dehydrogenase-2 (ALDH2) and central position gene Phosphatidic Acid Phosphatase Type 2C (PPAP2C), both of which are targets of miR1. Both of these target genes were differentially expressed in LIHC data set 1 analysis. The knockdown of PPAP2C decreases cancer proliferation in *in vitro* transformed stem cells, and drugs targeting PPAP2C may prove useful in treating LIHC [28]. Additionally, abnormal expression of ALDH2 was closely associated with increased risk of LIHC [29]. MiR-1, a key regulator of ALDH2 and PPAP2C in path:00561_1, is involved in the development of many cancers, including bladder, liver, lung, prostate, and colorectal cancer [30–34]. Moreover, miR-1 can simultaneously inhibit growth and promote differentiation of hepatocytes by inhibiting expression of its target genes [34, 35]. Even low levels of miR-1 are sufficient to partially suppress the activity of oncogenic target genes in cancer cells [32]. Though miR-1 expression did not change dramatically, Subpathway-GMir was still able to identify it as a key miRNA in an LIHC-relevant pathway.

The third subpathway, path00030_1, is part of the pentose phosphate metabolic pathway (PPP) and also overlaps extensively with the glycolysis/gluconeogenesis pathway (Figure 3C). Path:00030_1 was identified using Subpathway-GMir at *FDR* = 6.28E-09, and neither ORA (*FDR* = 0.18) nor the Kretschmann *et al.* (*FDR* = 0.56) method detected PPP. Furthermore, the Kretschmann *et al*. method also failed to detect the glycolysis/gluconeogenesis pathway (*FDR* = 0.14). Decreases in oxidative phosphorylation upregulates glycolysis in cancer cells [36], so the energy-switching role of path:00030_1 might contribute to LIHC development. The three miR-520 family members (miR-520a-3p, miR-520b, and miR-520e) in path:00030_1 together inhibited the core gene Platelet Isoform of Phosphofructokinase (PFK). Both the differential miR-520 family and target gene PFK can disrupt metabolic processes by triggering switches between the PPP and glycolysis pathways in LIHC. Hasawi et al. found that PFK catalyzes the conversion of fructose-6P-phosphotransferase to diphosphate-fructose-6-phosphate-1-phosphotransferase, which increases glycolysis and contributes to cancer progression [37]. The miR-520 family also inhibits the expression of PFK, upregulating glycolysis and promoting the proliferation and progression of cancer cells [38]. Transitions between PPP and glycolysis are also important in glioblastoma stem-like cells, and PFK expression is elevated during PPP and reduced during glycolysis [36, 39]. Our Subpathway-GMir results suggest that PPP and the glycolysis/gluconeogenesis pathway are likely important in LIHC as well.

### Dissecting key risk miRNAs in STAD-relevant metabolic subpathways

We had evaluated the effectiveness of Subpathway-GMir in identifying phenotype-associated metabolic pathways, we further dissected the mechanisms of miRNAs in mediating crosstalking genes from multiple subpathways. We then used Subpathway-GMir to analyze the STAD data set using 5633 differential genes and 344 differential miRNAs. We identified 20 significant subpathways (*FDR* < 0.01) associated with 20 complete pathways, of which 18 involved cancer-related miRNAs (Supplementary Table S2). Furthermore, 14 of these pathways are involved in carcinoma genesis. Additionally, up to 10 of the miRNA-mediated pathways were not detected by either ORA or the Kretschmann *et al*. method (Supplementary Figure S2).

**Figure 4:**
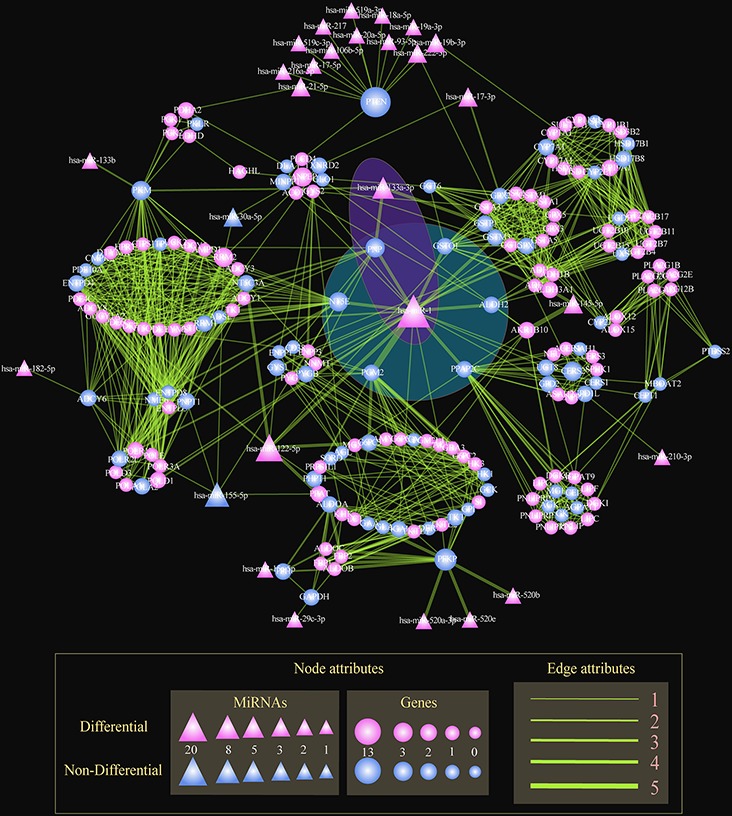
The global regulatory network of miRNAs in STAD Nodes represent genes or miRNAs within subpathways. Circle and triangle nodes represent genes and miRNAs, respectively. The gene (miRNA) node size is proportional to the number of linked miRNAs (genes). Differential nodes are colored red and non-differential nodes are colored blue. Edges represent the linking of two nodes involved the common subpathways. The line width is proportional to the number of subpathways involving the two linked two nodes. The blue and purple shaded regions show miR-1 and the miR-1/miR-133a cluster, respectively, mediating multiple subpathways.

To identify key miRNAs common to multiple subpathways, we built the STAD-relevant regulatory network by merging the 18 miRNA-mediated metabolic subpathways identified using Subpathway-GMir method (Figure 4). The hub miR-1 had up to 20 target genes, of which 6 genes were associated with cancers and involved at least three subpathways (Figure 4: The blue shaded region). The knockdown of one of those genes, PPAP2C, decreases the proliferation of cancer cells [28]. Two more of these target genes, NT5E and PNP, are candidate biomarkers for malignant melanoma [40]. Increased activity of the PGM2 target gene can facilitate immortalization of primary mouse embryo fibroblasts [41]. Finally, gene targets GSTO1 and ALDH2 are associated with breast, ovarian, and esophageal squamous cell cancer [42, 43]. Together, this indicates that miR-1 might disturb multiple subpathways by affecting crosstalking genes, ultimately promoting the development of STAD.

Multiple miRNAs together also regulated multiple subpathways in the STAD-relevant regulatory network. As shown in the purple shaded region in Figure 4, miR-1 and miR-133a-3p targeted the key gene PNP; this miRNA pair also regulated the Purine, Pyrimidine and Nicotinate and nicotinamide metabolic subpathways. MiR-133a-3p, a second regulator, clusters with miR-1 to inhibit genes involved in multiple cancer-relevant subpathways. When such clusters form between miRNAs separated by less than 50Kb, high correlations in expression result [44]. Correlations between miR-1/miR-133a have been observed in many cancers, including maxillary sinus squamous cell carcinoma (MSSCC), head and neck SCC, renal cell carcinoma, esophageal SCC, prostate cancer, colorectal cancer, and rhabdomyosarcoma [45–47]. The oncogenic PNP gene, an important metabolic enzyme which may be a therapeutic target in malignant lymphoproliferative diseases [48], is regulated by the miR-1/miR-133a cluster. Nohata *et al*. demonstrated that downregulating the miR-1/miR-133a cluster can significantly upregulate PNP and enhance carcinogenesis in MSSCC [45]. Additionally, decreasing miR-1/miR-133a cluster expression promotes oncogenesis by increasing PNP expression in prostate cancer [49]. These results suggest that Subpathway-GMir is able to identify miRNA clusters and their target genes in a variety of other pathways.

### Applying T2D data set

To examine the utility of Subpathway-GMir for diseases other than cancers, 131 T2D-related miRNAs and 2391 differential genes were used to identify T2D-relevant metabolic pathways. We identified 10 subpathways associated with 10 complete pathways using Subpathway-GMir method (*FDR* < 0.01). Seven of these complete pathways involved the development and progression of T2D (Supplementary Table S3). However, no and only three pathways were detected using the Kretschmann *et al*. and ORA method, respectively (*FDR* < 0.01). Moreover, 2 of 3 ORA pathways were also identified with Subpathway-GMir and verified by previous research (Supplementary Figure S3). The purine metabolism pathway was most significant among those identified by Subpathway-GMir (*FDR* < 1.00E-16) but not by ORA or the Kretschmann *et al*. method. Research suggests that the six purine metabolites of purine metabolism pathway might be useful in monitoring the progression, and evaluating the treatment, of T2D [50, 51]. These results indicate that Subpathway-GMir is useful for identifying metabolic pathways related to diseases other than cancers.

### Reproducibility and robustness analyses

#### Reproducibility analysis

**Figure 5:**
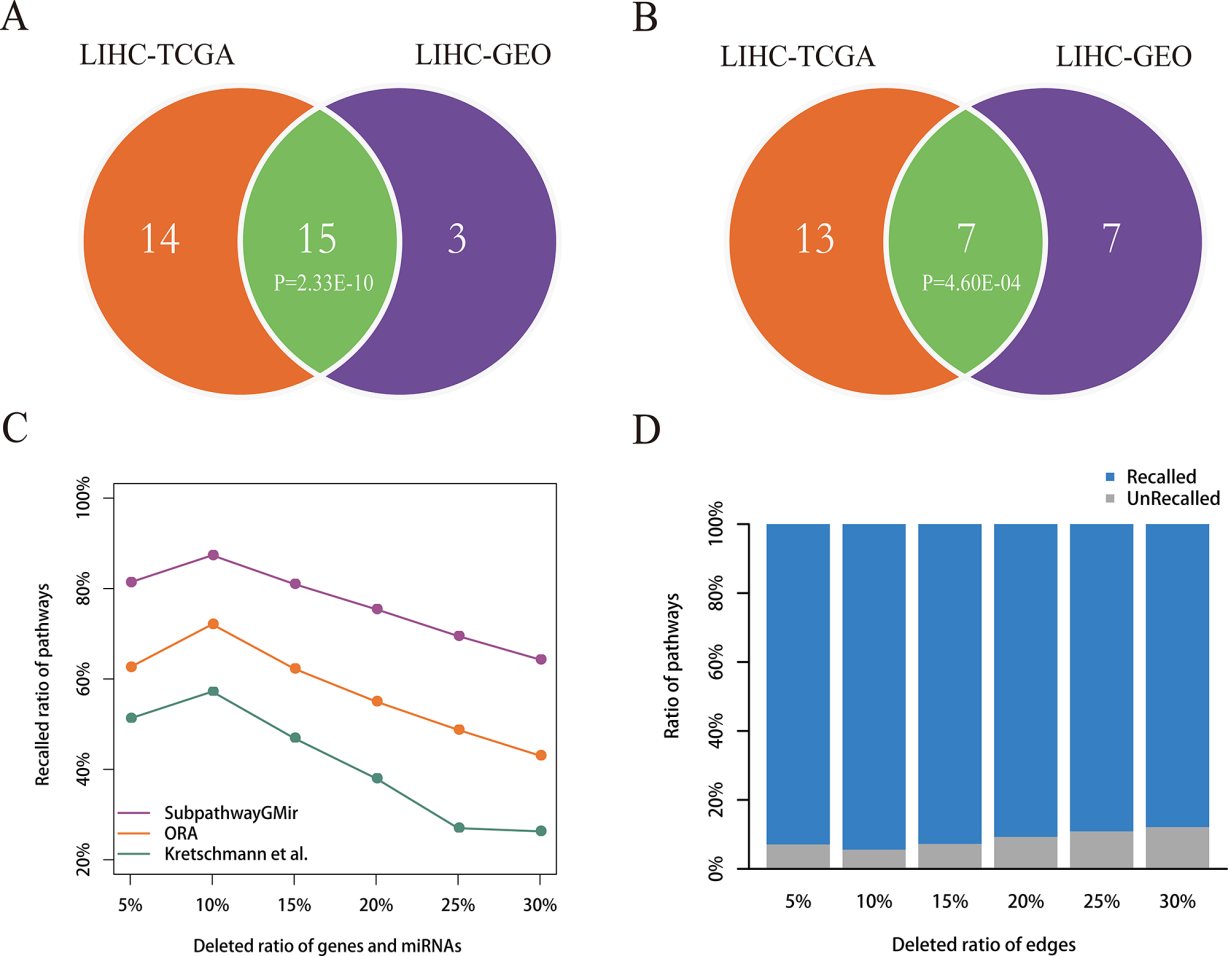
Reproducibility and robustness analyses **A–B.** Reproducibility analysis. Venn diagram A depicts all significant pathways identified by Subpathway-GMir in LIHC data sets 1 and 2. Venn diagram B depicts the significant pathways identified by Subpathway-GMir but not by both ORA or the Kretschmann *et al*. method in LIHC data sets 1 and 2. **C–D.** Robustness analysis. Figure C shows the mean ratio of recalled pathways using Subpathway-GMir, ORA and the Kretschmann *et al*. method after randomly deleting *N*% of genes and miRNAs from the corresponding profiles, where *N* = 5, 10, …, 30. Figure D shows the mean ratio of recalled pathways using Subpathway-GMir after randomly deleting *N*% of the edges in each RMPG, where *N* = 5, 10, …, 30.

To confirm that the results obtained using Subpathway-GMir were reproducible, we used it to analyze a second LIHC data set. Subpathway-GMir identified 18 significant subpathways associated with 18 complete pathways using 81 differential miRNAs and 2427 differential genes from LIHC data set 2 (*FDR* < 0.01, Supplementary Table S4). Among these, 14 pathways were identified only by Subpathway-GMir and not ORA or the Kretschmann *et al*. method (Supplementary Figure S4). We used a hypergeometric test to evaluate the significance of shared pathways between the two LIHC data sets (Table 1). The results showed that LIHC data set 1 and 2 shared 15 Subpathway-GMir pathways (*P* = 2.33E-10), and 7 of these pathways, undetected by both ORA and the Kretschmann *et al*. method, were specifically identified by Subpathway-GMir (*P* = 4.60E-04) method (Figure 5A–5B). This indicated that the results obtained using Subpathway-GMir were reproducible. Of the 15 pathways in both data sets, 11 are known to be associated with cancers; examples include the glycerophospholipid [52], fatty acid [53] and inositol phosphate metabolic pathways [54, 55]. 83% of LIHC data set 2 pathways were also identified in LIHC data set 1. We then evaluated whether this reproducibility was due to common differential genes and miRNAs that regulated the shared subpathways. Surprisingly, only 30% of the differential genes and 5% of the differential miRNAs identified in LIHC data set 1 were also identified in LIHC data set 2. Though there was a big difference in differential genes and miRNAs between two LIHC data sets, the findings identified by Subpathway-GMir still showed the well reproducibility.

**Table 1:** Comparison of pathways identified by subpathway-GMir, ORA and the kretschmann *et al*. method for the two LIHC data sets

PathwayName	TCGA Data Set			GEO Data Set		
	Subpathway-GMir	ORA	Kretschmann et al.	Subpathway-GMir	ORA	Kretschmann et al.
Glycolysis / Gluconeogenesis	√	√				
Purine metabolism	√	√		√	√	
Pyrimidine metabolism	√	√		√	√	
Inositol phosphate metabolism	√		√	√		
Glycerophospholipid metabolism	√	√		√		
Metabolism of xenobiotics by cytochrome P450	√	√		√		
Lysine degradation	√	√				
Arachidonic acid metabolism	√					
Fatty acid metabolism	√	√		√		
Glycerolipid metabolism	√			√		
Retinol metabolism	√			√		
Fructose and mannose metabolism	√					
Drug metabolism - cytochrome P450	√					
Sphingolipid metabolism	√			√		
Pentose phosphate pathway	√					
Steroid hormone biosynthesis	√			√		
Tryptophan metabolism	√			√		
Starch and sucrose metabolism	√					
Arginine and proline metabolism	√	√		√		
Ether lipid metabolism	√					
Alanine, aspartate and glutamate metabolism	√			√	√	
Pyruvate metabolism	√					
beta-Alanine metabolism	√					
Tyrosine metabolism	√					
Valine, leucine and isoleucine degradation	√			√		
Glutathione metabolism	√			√		
Glycine, serine and threonine metabolism	√					
Galactose metabolism	√					
Histidine metabolism	√					
One carbon pool by folate				√	√	
Aminoacyl-tRNA biosynthesis				√		
Butanoate metabolism				√		

Note: The bold pathways were verified that associated with LIHC.

#### Robustness analysis

To test the stability of Subpathway-GMir, we evaluated its performance after randomly introducing noise in differential molecular sets and RMPGs. We first randomly deleted the expressions of *N*% genes and miRNAs from corresponding profiles. For each parameter *N* (= 5, 10, …, 30), we repeated this deletion process 1000 times to obtain 1000 randomly-generated profiles of genes and miRNAs, and then produced 1000 differential molecular lists. Subpathway-GMir, ORA, and the Kretschmann *et al*. method were used separately to obtain 1000 pathway lists from these 1000 differential molecular lists. For original 29, 8, 1 identified pathways as the reference sets, we compared the mean ratio of the reference pathways recalled from 1000 random pathway lists (*FDR* < 0.01), the results can be shown in Figure 5C. As the deletion ratio was increased, the performance of all three methods declined, but Subpathway-GMir consistently had higher recalled ratios than the other methods. Specifically, the recalled ratio for Subpathway-GMir was 87% when deletion ratio was 10%, whereas only 72% and 57% were recalled using ORA and the Kretschmann *et al*. method, respectively. When the deletion ratio was increased to 30%, the recalled ratio for Subpathway-GMir was 64%, while it was less than 50% for both ORA and the Kretschmann *et al*. method. Then we broaden the significance threshold of pathways, we found that our Subpathway-GMir method still produced better performance than both ORA and the Kretschmann *et al*. method when setting *FDR* < 0.05 (Supplementary Figure S5).

We next tested the performance of Subpathway-GMir by randomly deleting a percentage of the edges within each RMPG. For each deletion percentage (*N* = 5, 10, …, 30), we repeated the deletion process 1000 times to obtain 1000 random RMPG lists. Then we performed the Subpathway-GMir method using 3751 differential molecules for each random RMPG list as pathway background, and we obtained 1000 random pathway lists. As the same, we calculated the mean ratio of recall for 29 reference pathways out of the 1000 random pathway lists (Figure 5D). As the deletion percentage increased, the recalled ratio declined slightly. Subpathway-GMir displayed the best performance when the deletion ratio was 10%, recalling more than 94% of the pathways. When the deletion ratio was increased to 30%, the recalled ratio was 88%. In summary, the above results suggested that the pathways identified by Subpathway-GMir was robust in resisting the disturbance of differential molecular sets and RMPGs.

## DISCUSSION

MiRNAs generally disturbed metabolic pathways by inhibiting the expression of their target genes. To locate miRNA-mediated key subregions within complete pathways and explore the potential regulatory mechanisms of miRNAs and genes, we developed Subpathway-GMir method.

The Subpathway-GMir method has several advantages. First, the inclusion of both genes and miRNAs in reconstructed RMPGs, which is more reflective of real biological networks, likely contributed to the improvements in pathway detection seen with Subpathway-GMir. For example, the larger AAM pathway, of which LIHC-associated path:00590_1 is a part, was not detected by ORA and Kretschmann *et al*. method. This detection failure may be due to the neglect of differential miRNAs and genes, respectively, in those methods. Second, pathway topology was considered when locating candidate subregions, which was better to reflect the transmission of disease signals. Both PPP and glycolysis pathway, as the whole of Path:00030_1, are important for switching the metabolic processes in LIHC. However, the ORA and Kretschmann *et al*. method, independent of pathway topologies, failed to detect PPP pathway. Third, we set up two flexible parameters *n* and *s* to locate the candidate subpathways. Setting *n* = 1 requires that there is no more than one non-differential molecule between any two differential molecules, which makes disease signals stronger and the transmission more direct. The parameter *s* = 10 increases the number of differential molecules included in candidate subpathways, with the result that the subpathways may be sufficient to dysregulate the complete pathway of which they are a part. Therefore, we can identify miR-1 as a key miRNA in an LIHC-relevant pathway though the expression level of it was weakly changed.

The upstream regulators of metabolic pathways, miRNAs generally regulated the complete pathway by inhibiting their downstream targets within key subpathways. To further investigate the roles of cancer-related miRNAs, we constructed the global STAD-relevant regulatory network. The hub miRNAs, as a central part of network, were important to the stability of the global metabolic system. Thus we focused on the miRNAs that impact the largest numbers of metabolism genes. We found that miR-1 targeted most genes in network, and the six of them were cancer-relevant and crosstalked by multiple subpathways. Moreover, miR-133a-3p can cluster with miR-1, in manifold cancers, to inhibit the crosstalking genes from multiple subpathways. It suggests that Subpathway-GMir can be used to identify individual miRNA, miRNA clusters and the genes they target in multiple pathways, thereby aiding in the development of novel therapies for many different cancers.

The flexibility of the Subpathway-GMir model makes it useful in evaluating many pathways related to diseases other than cancers, as demonstrated in T2D data set analysis. The highly reproducible nature of miRNA and gene pathways identified using Subpathway-GMir, by comparing two different LIHC data set results, also suggests that it will be a useful tool for understanding metabolic regulation in many diseases. Subpathway-GMir method was implemented as a freely available R-based tool. The users can flexibly choice the environment variables, supporting one of six species, to identify organism-specific metabolic subpathways.

## MATERIALS AND METHODS

### Materials

#### Experimentally verified miRNA-target interactions

We collected experimentally verified miRNA-target interactions from miRTarBase [12], mir2Disease [13], miRecords (V4.0) [11] and TarBase (V6.0) [10] databases. These interactions were identified from research done in six species. After redundancy processing, 55146 miRNA-target interactions among 20186 genes and 1110 miRNAs were obtained as follows: 96 pairs from mir2Disease, 518 pairs from miRecords (V4.0), 26388 pairs from TarBase (V6.0), and 50381 pairs from miRTarBase. A total of 40990 human specific miRNA-target interactions involving 14653 genes and 579 miRNAs were obtained; 7630 of these pairs, involving 4376 genes and 371 miRNAs, had been verified by low-throughput experiments.

#### LIHC data set 1

The RNA-seq datasets of genes and miRNAs (quantile-normalized and background-corrected at level three) were downloaded from The Cancer Genome Atlas (TCGA) database (http://tcga-data.nci.nih.gov/). Here, we used reads per kilobase of exon per million fragments mapped (RPKM) and read count datasets. The miRNA expression profile included 100 cancer samples and 50 normal samples; the gene expression profile included 17 cancer samples and 9 normal samples. For each type of dataset, we used average expression for genes and miRNAs more than one value. To get differentially expressed genes and miRNAs, we applied fold change (FC) method to the RPKM data set and the edgeR [56] method to the read count data set. Genes and miRNAs were considered differentially expressed when the edgeR method false discovery rate (*FDR*) was < 0.05 and the FC ratio |log2(*FC*)| > 1. We identified 3357 differentially expressed genes and 268 differentially expressed miRNA precursors (pre-miRNAs). Finally, we converted 268 pre-miRNAs to 394 mature miRNAs (mat-miRNAs) basing on the corresponding relationships between pre-miRNAs and mat-miRNAs from the miRBase [57] database.

#### LIHC data set 2

Processed expression matrices of genes and miRNAs were extracted from Gene Expression Omnibus (GEO) database (http://www.ncbi.nlm.nih.gov/geo/). The miRNA expression matrix included 68 cancer samples and 21 normal samples (GSE36915) [58]; the gene expression matrix included 6 cancer samples and 6 normal samples (GSE46408) [59]. We considered genes and miRNAs differentially expressed when the significance analysis of microarrays (SAM) [60] method had *FDR* < 0.05 and FC ratio |log2(*FC*)| > 1. We identified 2427 differentially expressed genes and 81 differentially expressed mat-miRNAs.

#### STAD data set

The RPKM and read count datasets of genes and miRNAs were downloaded and processed the same as for LIHC data set 1. We extracted sample-matched gene and miRNA profiles in this case, including 231 cancer samples and 33 normal samples. Using the same process as for LIHC data set 1, we identified 5633 differential genes and 229 differential pre-miRNAs corresponding to 344 mat-miRNAs.

#### T2D data set

The gene expression profile data was originally analyzed and processed by *Jain et al*. (GSE29221) [61] and included 12 diabetic and non-diabetic samples each. We obtained 2391 differential expressed genes using the SAM method with an *FDR* < 0.1 and FC ratio |log2(*FC*)| > 1. Searches were conducted for the T2D-related risk miRNAs in titles or abstracts using PubMed with the following terms: (‘miRNA’, ‘type 2 diabetes’) or (‘microRNA’, ‘type 2 diabetes’). A total of 131 miRNAs from 66 sources were obtained (Supplementary Table S5).

### Methods

**Figure 6:**
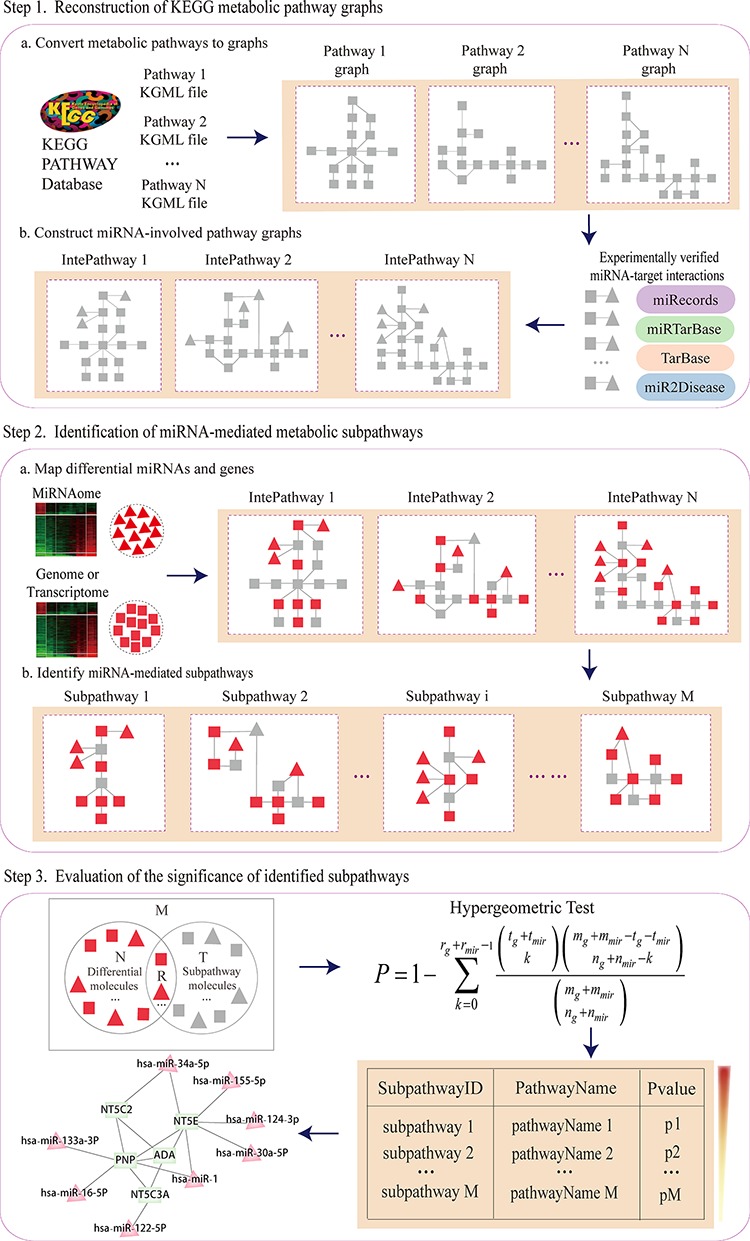
The pipeline overview of Subpathway-GMir

Subpathway-GMir implements the credible reconstruction of KEGG metabolic pathways by embedding miRNAs with target genes verified by low-throughput experiments. MiRNA-mediated metabolic subpathways are identified by topologically analyzing the positions and cascade regions of condition-specific genes and miRNAs. Furthermore, Subpathway-GMir has been implemented as a freely available R package at http://cran.r-project.org/web/packages/SubpathwayGMir/. It can support the identification of miRNA-mediated metabolic subpathways in six species, abbreviated as follows: cel (*Caenorhabditis elegans*), dme (*Drosophila melanogaster*), dre (*Danio rerio*), hsa (*Homo sapiens*), mmu (*Mus musculus*) and rno (*Rattus norvegicus*). Users can alter the environment variables to identify organism-specific metabolic subpathways mediated by miRNAs. The pipeline overview is depicted in Figure 6. It contains three main components: (i) By converting KEGG metabolic pathways into graphs with genes as nodes, we build reconstructed KEGG metabolic pathway graphs (RMPGs) that integrate miRNA-target interactions supported by low-throughput experiments; (ii) It maps condition-specific genes and miRNAs into RMPGs and identifies miRNA-mediated metabolic subpathways based on the “lenient distance” similarity method; (iii) It evaluates the significance of candidate subpathways using the hypergeometric method. The details of these processes are described below.

#### Reconstructed KEGG metabolic pathway graphs

The reconstructed KEGG metabolic pathway graphs contained both genes and miRNAs, replicating real biological pathways. We first extracted 152 KEGG metabolic pathway structures and converted them into undirected graphs with genes as nodes and compounds as edges using our previously developed R package “*iSubpathwayMiner*” [15]. We next searched each miRNA with at least one target gene verified in low-throughput experiments, and determined whether each converted pathway graph contained its verified targets or not. For each pathway, we then embedded the miRNA node into the pathway and linked it to its verified targets. Finally, we obtained the RMPGs, which incorporated miRNA nodes and miRNA-target interaction edges.

#### Locate metabolic subpathways mediated by miRNAs

We located miRNA-mediated metabolic subpathways using the “lenient distance” similarity method [16]. We first mapped differentially expressed genes and miRNAs into RMPGs as signature nodes, then computed the shortest path between any two signatures. For each signature pair, we placed the two signatures, and the molecules contained in the shortest path (the length of which was set to no longer than *n*) between them, into the same candidate node. We then located each candidate node set in the pathway graph and extracted the corresponding subregion. We finally defined the subregion as a subpathway where the number of nodes was no less than *s*. The *n* and *s* parameters control the intensity of disease signals and the size of candidate subpathways, respectively. The default parameters *n* = 1 and *s* = 10 were used here.

#### Evaluate the statistic significance of located subpathways

To evaluate whether located subpathways were dysregulated in disease conditions comparing to chance, we used the hypergeometric method to test their statistical significance. The following formula was used to calculate *P*-value for the enrichment significance of subpathways:

$$P=1-\sum_{k=0}^{r_{g}+r_{m,i,r}-1}\frac{\left(\begin{array}{l} t_{g}+t_{m,i,r}\\ k \end{array}\right)\left(\begin{array}{l} m_{g}+m_{m,i,r}-t_{g}-t_{m,i,r}\\ n_{g}+n_{m,i,r}-k \end{array}\right)}{\left(\begin{array}{l} m_{g}+m_{m,i,r}\\ n_{g}+n_{m,i,r} \end{array}\right)}$$

In above formula, *m_g_ (m_mir_)* was the number of genes (miRNAs) in entire genome (miRNAome), and *n_g_ (n_mir_)* was the number of differentially expressed genes (miRNAs), of which *r_g_ (r_mir_)* genes (miRNAs) participated in the subpathway containing *t_g_* genes (*t_mir_* miRNAs).

## SUPPLEMENTARY FIGURES AND TABLES


