# Supplementary material for: Subpathway-GMir: identifying miRNA-mediated metabolic subpathways by integrating condition-specific genes, microRNAs, and pathway topologies
Source: Oncotarget. 2015 Oct 12;6(36):39151–64. doi: 10.18632/oncotarget.5341 (PMC4770763; doi:10.18632/oncotarget.5341)
Supplement: Supplementary file 1 [file oncotarget-06-39151-s001.pdf]

SUPPLEMENTARY FIGURES AND TABLES

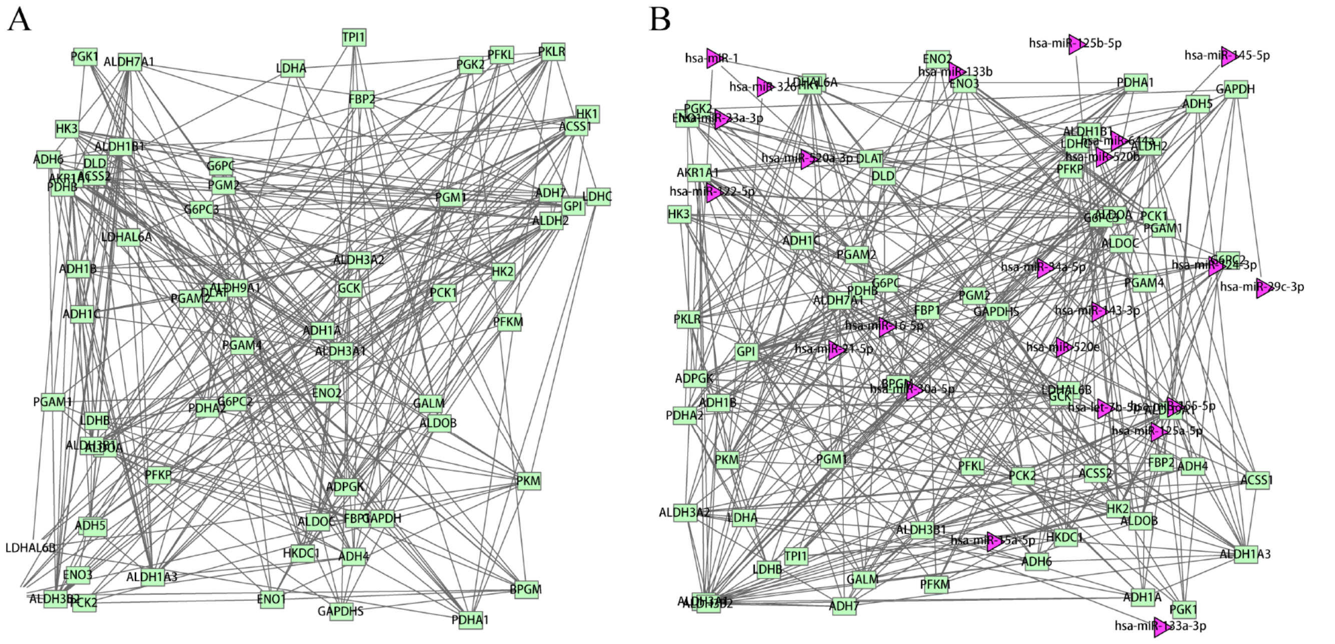

Supplementary Figure S1: The "Glycolysis/Gluconeogenesis" metabolic pathway graph before and after reconstructing. The rectangle and triangle nodes represent genes and miRNAs, respectively. **A.** The graph before reconstructing. **B.** The graph after reconstructing.

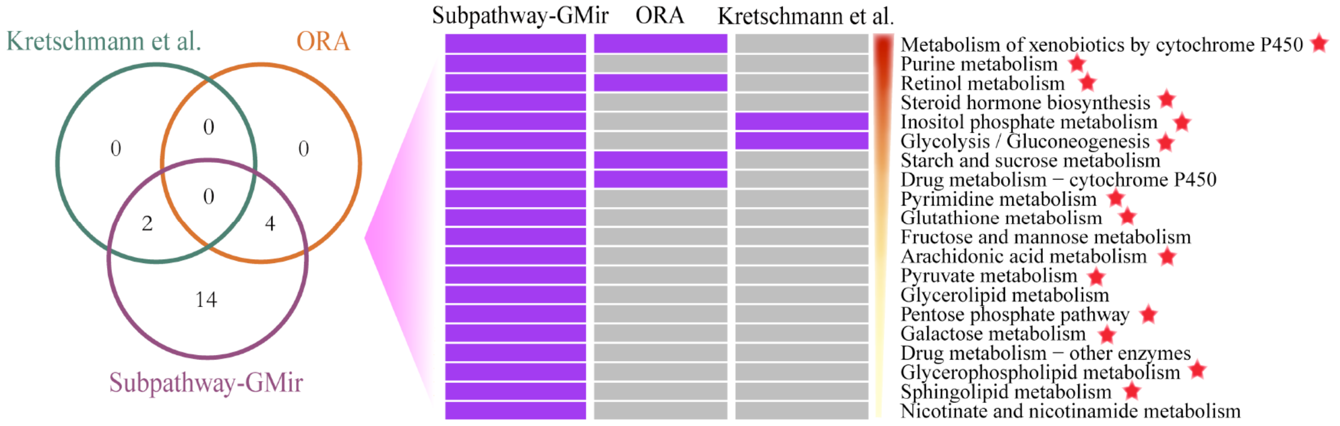

Supplementary Figure S2: Comparison of pathways identified by Subpathway-GMir, ORA and the Kretschmann *et al*. method in STAD data set (*FDR* < 0.01). Venn diagram depicting the overlap of pathways. These pathways were sorted by statistical significance (using Subpathway-GMir) in descending order. Each row represents a pathway and each column represents a method. The purple and grey grid represents that the pathway was or wasn't identified by the method, respectively. Pathways supported by scientific studies are marked with red stars.

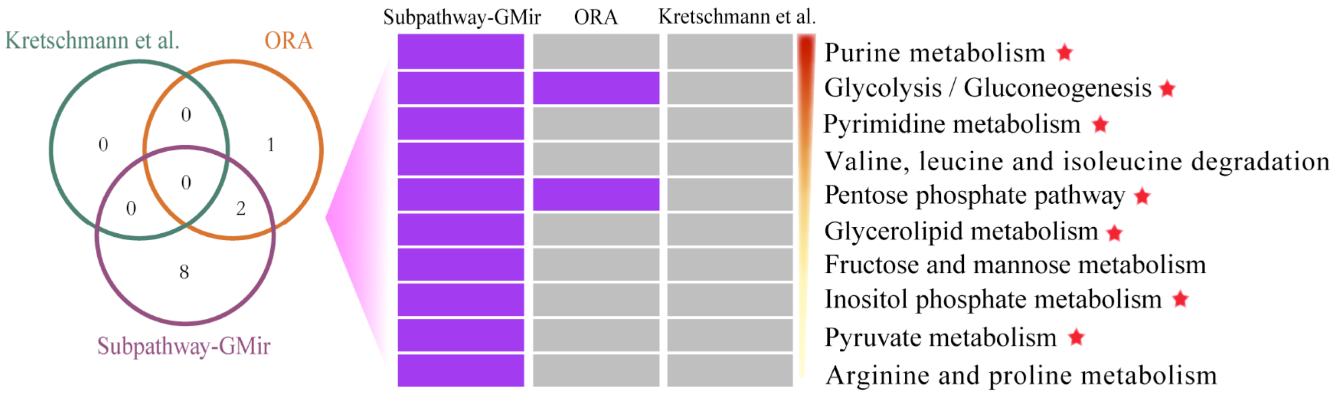

Supplementary Figure S3: Comparison of pathways identified by Subpathway-GMir, ORA and the Kretschmann *et al*. method in T2D data set (*FDR* < 0.01). Venn diagram depicting the overlap of pathways. These pathways were sorted by statistical significance (using Subpathway-GMir) in descending order. Each row represents a pathway and each column represents a method. The purple and grey grid represents that the pathway was or wasn't identified by the method, respectively. Pathways supported by scientific studies are marked with red stars.

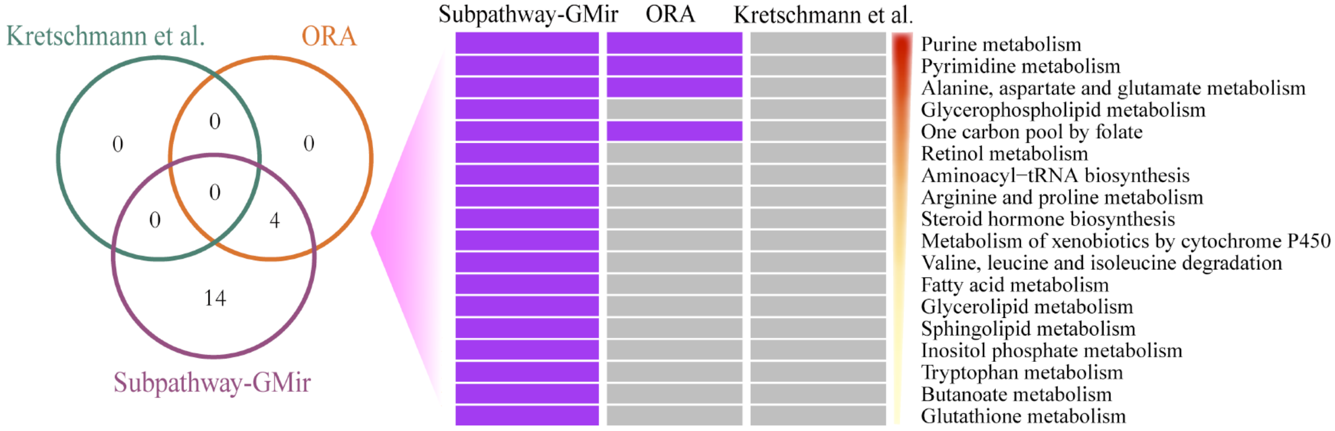

Supplementary Figure S4: Comparison of pathways identified by Subpathway-GMir, ORA and the Kretschmann *et al*. method in LIHC data set 2 (*FDR* < 0.01). Venn diagram depicting the overlap of pathways. These pathways were sorted by statistical significance (using Subpathway-GMir) in descending order. Each row represents a pathway and each column represents a method. The purple and grey grid represents that the pathway was or wasn't identified by the method, respectively.

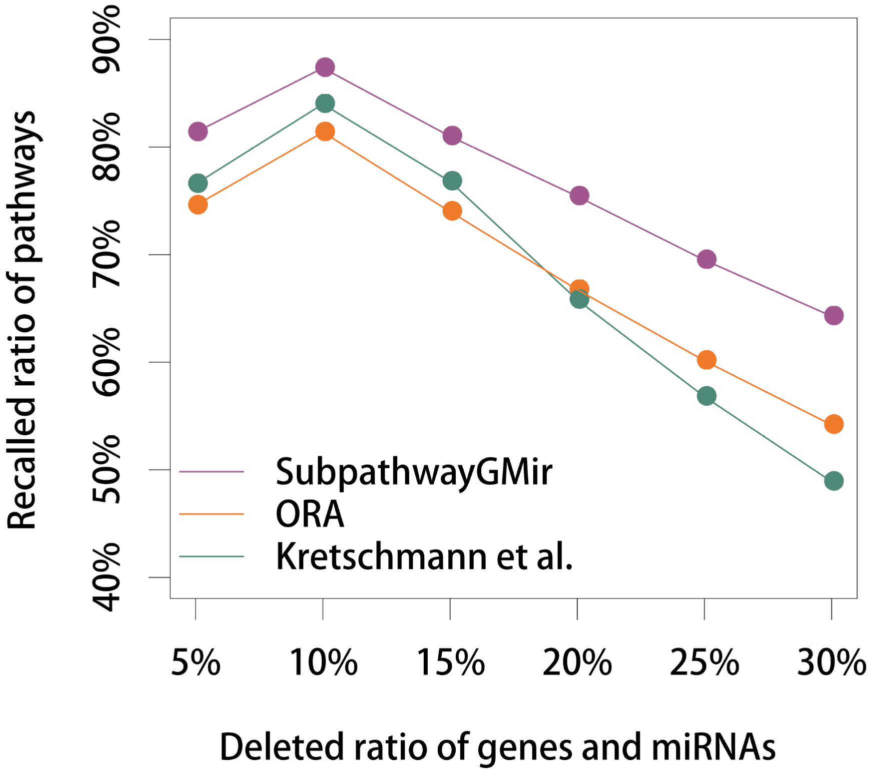

Supplementary Figure S5: The mean ratio of recalled pathways using Subpathway-GMir, ORA and the Kretschmann *et al*. method (*FDR* < 0.05).

Supplementary Table S1: Subpathways identified using subpathway-GMir in LIHC data set 1

Pathway Id	Pathway Name	FDR	Possible relations to cancers	Cancer-related miRNAs	Reference
path:00010_1	Glycolysis / Gluconeogenesis	<1.00E-16	MiR-520a/b/e regulate glycolysis in HCC	miR-34a-5p;miR-21-5p; miR-133a-3p;miR-326; miR-29c-3p;miR-520b; miR-520e;miR-125b-5p	[1]
path:00230_1	Purine metabolism	<1.00E-16	Transformation and progression in hepatomas	miR-34a-5p;miR-24-3p; let-7c-5p;miR-182-5p; miR-133a-3p;miR-326	[2]
path:00240_1	Pyrimidine metabolism	<1.00E-16	Transformation and progression in hepatomas	miR-34a-5p;miR-24-3p; let-7c-5p;miR-133a-3p	[2]
path:00562_1	Inositol phosphate metabolism	<1.00E-16	MiR-21 mediate the increase of cell proliferation and invasion in HCC MiR-221&222 enhance tumorigenicity in HCC	miR-217;miR-216a-5p; miR-214-3p;miR-21-5p; miR-519a-3p;miR-519d-3p; miR-19a-3p;miR-141-3p; miR-18a-5p;miR-221-3p; miR-222-3p;miR-93-5p; miR-519c-3p;miR-34a-5p; miR-205-5p;miR-184	[3, 4]
path:00564_1	Glycerophospholipid metabolism	<1.00E-16	Drug target against cancer	miR-338-3p;miR-34a-5p	[5]
path:00980_1	Metabolism of xenobiotics by cytochrome P450	3.86E-14	Poor prognosis of HCC	miR-513a-3p;miR-133a-3p	[6]

(*Continued*)

Pathway Id	Pathway Name	FDR	Possible relations to cancers	Cancer-related miRNAs	Reference
path:00310_1	Lysine degradation	1.18E-12	—	miR-34a-5p;miR-21-5p	—
path:00590_1	Arachidonic acid metabolism	4.07E-12	MiRNA-101 promotes apoptosis and suppress in HCC<br>Cox-2 promotes the growth of HCC cell	miR-137;miR-101-3p; miR-338-3p	[7, 8]
path:00071_1	Fatty acid metabolism	1.63E-11	Significantly altered fatty acid-related metabolic pathways in HCC	miR-34a-5p	[9]
path:00561_1	Glycerolipid metabolism	1.52E-10	MiR-125b could inhibit cell proliferation, cell cycle progression and metastasis of HCC cells	miR-125b-5p;miR-34a-5p	[10]
path:00830_1	Retinol metabolism	2.72E-10	The high prediagnostic serum level of retinol is associated with a decreased risk of HCC	miR-34a-5p;miR-125b-5p	[11, 12]
path:00051_1	Fructose and mannose metabolism	8.50E-10	—	miR-34a-5p;miR-125b-5p; miR-520b;miR-520e	—
path:00982_1	Drug metabolism - cytochrome P450	8.88E-10	—	miR-513a-3p;miR-133a-3p	—
path:00600_1	Sphingolipid metabolism	4.79E-09	Chemoprevention target of HCC; Tumor growth of hepatocellular carcinoma	miR-221-3p;miR-222-3p; miR-125b-5p	[13–15]
path:00030_1	Pentose phosphate pathway	6.28E-09	PPP activity is elevated in rapidly proliferating tumor cells	miR-34a-5p;miR-520b; miR-520e	[16, 17]
path:00140_1	Steroid hormone biosynthesis	8.86E-09	Carcinogenesis in HCC	miR-199a-5p	[18, 19]
path00380_1	Tryptophan metabolism	2.54E-08	—	miR-34a-5p	—
path:00500_1	Starch and sucrose metabolism	2.54E-08	—	miR-125b-5p;miR-34a-5p	—
path:00330_2	Arginine and proline metabolism	2.54E-08	—	miR-34a-5p;miR-24-3p	—
path:00565_1	Ether lipid metabolism	2.54E-08	—	miR-338-3p	—
path:00250_1	Alanine, aspartate and glutamate metabolism	3.63E-08	—	miR-34a-5p	—
path:00620_1	Pyruvate metabolism	1.24E-07	Metabolism and tumour growth	miR-21-5p;miR-133a-3p; miR-326;miR-34a-5p	[20, 21]
path:00410_1	beta-Alanine metabolism	1.64E-07	—	miR-34a-5p;miR-24-3p	—

(*Continued*)

Pathway Id	Pathway Name	FDR	Possible relations to cancers	Cancer-related miRNAs	Reference
path:00350_1	Tyrosine metabolism	1.14E-06	Pathogenesis of HCC	—	[22]
path:00280_2	Valine, leucine and isoleucine degradation	1.58E-06	—	miR-34a-5p	—
path:00480_1	Glutathione metabolism	3.83E-06	The growth of liver cancer cells	miR-183-5p;miR-513a-3p; miR-133a-3p	[23]
path:00260_1	Glycine, serine and threonine metabolism	2.03E-05	—	—	—
path:00052_1	Galactose metabolism	2.03E-05	The treatment of HCC	miR-34a-5p;miR-125b-5p	[24, 25]
path:00340_2	Histidine metabolism	2.03E-05	Carcinogenesis	miR-24-3p;miR-34a-5p	[26]

Supplementary Table S2: Subpathways identified using subpathway-GMir in STAD data set

Pathway Id	Pathway Name	FDR	Possible relations to cancer	Cancer-related miRNAs	Reference
path:00980_1	Metabolism of xenobiotics by cytochrome P450	2.22E-15	Development of STAD	miR-122-5p;miR-133a-3p; miR-1;miR-145-5p	[27, 28]
path:00230_1	Purine metabolism	1.89E-14	Development of STAD	miR-1;miR-182-5p;miR-122-5p; miR-133a-3p;miR-133b	[29]
path:00830_1	Retinol metabolism	3.12E-13	Prognosis of STAD	—	[30]
path:00140_1	Steroid hormone biosynthesis	9.63E-13	Apoptosis and Prognosis	miR-1;miR-19b-3p;miR-122-5p	[31, 32]
path:00562_2	Inositol phosphate metabolism	9.86E-12	Cancer aggressiveness	miR-217;miR-17-5p;miR-216a-5p; miR-21-5p;miR-519a-3p;miR-19a-3p; miR-19b-3p;miR-18a-5p;miR-20a-5p; miR-222-3p;miR-106b-5p;miR-93-5p; miR-519c-3p;miR-1	[33]
path:00010_1	Glycolysis / Gluconeogenesis	1.46E-11	Prognosis	miR-122-5p;miR-1;miR-145-5p; miR-29c-3p;miR-15a-5p; miR-520b;miR-520e	[34]
path:00500_1	Starch and sucrose metabolism	4.02E-11	—	miR-122-5p;miR-1	—
path:00982_1	Drug metabolism - cytochrome P450	1.49E-09	—	miR-145-5p;miR-122-5p; miR-133a-3p;miR-1	—
path:00240_1	Pyrimidine metabolism	1.88E-09	Differentiation of STAD	miR-1;miR-122-5p;miR-17-3p; miR-133a-3p	[35]
path:00480_1	Glutathione metabolism	1.86E-08	Development of STAD	miR-1;miR-17-3p;miR-122-5p; miR-133a-3p	[36, 37]

(*Continued*)

Pathway Id	Pathway Name	FDR	Possible relations to cancer	Cancer-related miRNAs	Reference
path:00051_1	Fructose and mannose metabolism	3.64E-08	—	miR-145-5p;miR-122-5p; miR-520b;miR-520e;miR-15a-5p	—
path:00590_1	Arachidonic acid metabolism	4.27E-08	Apoptosis of gastric cancer	miR-1;miR-17-3p	[38, 39]
path:00620_1	Pyruvate metabolism	6.46E-08	Growth of gastric cancer	miR-21-5p;miR-133b; miR-133a-3p;miR-145-5p;miR-1	[40, 41]
path:00561_1	Glycerolipid metabolism	7.24E-08	—	miR-1;miR-145-5p	—
path:00030_1	Pentose phosphate pathway	1.49E-07	Apoptosis of gastric cancer	miR-1;miR-122-5p; miR-520b;miR-520e	[42]
path:00052_1	Galactose metabolism	3.02E-07	Metastases	miR-145-5p;miR-1;miR-122-5p	[43]
path:00983_4	Drug metabolism - other enzymes	5.07E-07	—	—	—
path:00564_1	Glycerophospholipid metabolism	7.71E-06	The treatment of cancer	miR-1;miR-210-3p	[5]
path:00600_1	Sphingolipid metabolism	4.08E-05	Apoptosis, Progression and survival of gastric cancer	miR-1;miR-222-3p	[44, 45]
path:00760_1	Nicotinate and nico-tinamide metabolism	4.76E-04	—	miR-1;miR-122-5p;miR-133a-3p	—

Supplementary Table S3: Subpathways identified using subpathway-GMir in T2D data set

Pathway Id	Pathway Name	FDR	Possible relations to T2D	T2D-related miRNAs	Reference
path:00230_1	Purine metabolism	<1.00E-16	Progression and treatment	miR-320a;miR-133b	[46, 47]
path:00010_1	Glycolysis / Gluconeogenesis	1.04E-12	Gluconeogenesis is enhanced in T2D<br>The sustained gluconeogenesis produced the hallmark of T2D	miR-133b;miR-143-3p	[48, 49]
path:00240_1	Pyrimidine metabolism	2.12E-09	Progression and treatment	—	[50]
path:00280_1	Valine, leucine and isoleucine degradation	1.39E-08	—	—	—
path:00030_1	Pentose phosphate pathway	1.39E-08	Progression and treatment	—	[51]
path:00561_1	Glycerolipid metabolism	2.25E-08	Treatment of T2D	—	[52, 53]
path:00051_1	Fructose and mannose metabolism	9.07E-08	—	miR-143-3p	—
path:00562_1	Inositol phosphate metabolism	2.16E-07	Downregulated in T2D	miR-17-5p;miR-107;hsa-miR-184	[54]
path:00620_1	Pyruvate metabolism	6.75E-07	Development of T2D	miR-133b	[55, 56]
path:00330_1	Arginine and proline metabolism	3.21E-06	—	—	—

Supplementary Table S4: Subpathways identified using subpathway-GMir in LIHC data set 2

Pathway Id	Pathway Name	FDR
path:00230_1	Purine metabolism	<1.00E-16
path:00240_1	Pyrimidine metabolism	<1.00E-16
path:00250_1	Alanine, aspartate and glutamate metabolism	1.56E-11
path:00564_1	Glycerophospholipid metabolism	1.11E-08
path:00670_1	One carbon pool by folate	1.11E-08
path:00830_1	Retinol metabolism	1.11E-08
path:00970_1	Aminoacyl-tRNA biosynthesis	3.56E-08
path:00330_1	Arginine and proline metabolism	4.22E-08
path:00140_1	Steroid hormone biosynthesis	7.29E-08
path:00980_1	Metabolism of xenobiotics by cytochrome P450	7.29E-08
path:00280_1	Valine, leucine and isoleucine degradation	1.13E-07
path:00071_1	Fatty acid metabolism	1.52E-07
path:00561_1	Glycerolipid metabolism	1.52E-07
path:00600_1	Sphingolipid metabolism	1.78E-07
path:00562_1	Inositol phosphate metabolism	4.66E-07
path:00380_1	Tryptophan metabolism	1.42E-06
path:00650_1	Butanoate metabolism	1.42E-06
path:00480_1	Glutathione metabolism	3.46E-06

Supplementary Table S5: The T2D-related risk miRNAs

miRNA	PMID
hsa-miR-196a2	25557604
hsa-miR-107	25522185
hsa-miR-17-5p	25409512
hsa-miR-221	25409512
hsa-miR-124a	25408296
hsa-miR-375	25408296
hsa-let-7	25399420
hsa-miR-18a	25371752
hsa-miR-199a-3p	25180600
hsa-miR-342	25180600
hsa-miR-195	25138607
hsa-miR-29a	25138607
hsa-miR-103	25010252
hsa-miR-143	25010252
hsa-miR-483-3p	25010252
hsa-miR-23a	24981880

(*Continued*)

miRNA	PMID
hsa-miR-196a2	24972764
hsa-miR-143-3p	24970281
hsa-miR-126	24927146
hsa-miR-191	24795757
hsa-miR-378	24771406
hsa-miR-149	24757201
hsa-miR-130a	24750349
hsa-miR-375	24741571
hsa-miR-146a	24682535
hsa-miR-128a	24682535
hsa-miR-27a	24682535
hsa-miR-15a	24497980
hsa-miR-20	24497980
hsa-miR-21	24497980
hsa-miR-24	24497980
hsa-miR-29b	24497980
hsa-miR-126	24497980
hsa-miR-144	24497980
hsa-miR-150	24497980
hsa-miR-197	24497980
hsa-miR-223	24497980
hsa-miR-191	24497980
hsa-miR-320a	24497980
hsa-miR-486-5p	24497980
hsa-miR-28-3p	24497980
hsa-miR-140-5p	24478399
hsa-miR-142-3p	24478399
hsa-miR-222	24478399
hsa-miR-423-5p	24478399
hsa-miR-125b	24478399
hsa-miR-192	24478399
hsa-miR-195	24478399
hsa-miR-130b	24478399
hsa-miR-532-5p	24478399
hsa-miR-126	24478399
hsa-miR-126	24455723
hsa-miR-92a	24379347
hsa-miR-193b	24379347

(*Continued*)

miRNA	PMID
hsa-miR-126	24379347
hsa-miR-375	24366165
hsa-miR-377-5p	24352417
hsa-miR-628-3p	24352417
hsa-miR-3137	24352417
hsa-miR-143	24333576
hsa-miR-145	24333576
hsa-miR-138	24204780
hsa-miR-15b	24204780
hsa-miR-376a	24204780
hsa-miR-503	24204780
hsa-miR-187	24149837
hsa-miR-184	24109547
hsa-let-7a	24105413
hsa-let-7d	24105413
hsa-miR-146a	24023848
hsa-miR-106b	23954742
hsa-miR-34a	23828613
hsa-miR-96	23828613
hsa-miR-143	23812417
hsa-miR-21	23771797
hsa-miR-155	23616185
hsa-miR-150	23391324
hsa-miR-342	23391324
hsa-miR-126	23386708
hsa-miR-375	23372846
hsa-miR-223	23305783
hsa-miR-30c	21878751
hsa-miR-146a	21249428
hsa-miR-34a	20857148
hsa-miR-181d	20639500
hsa-miR-375	20467341
hsa-miR-375	20224724
hsa-miR375	20221699
hsa-miR-223	20080987
hsa-miR-375	25120598
hsa-miR-143-3p	24970281
hsa-miR-326	24937531

(*Continued*)

miRNA	PMID
hsa-miR-let-7a	24937531
hsa-miR-let-7f	24937531
hsa-miR-92a	24379347
hsa-miR-181a	22476949
hsa-miR-483-3p	22223106
hsa-miR-27a	23032062
hsa-miR-320a	23032062
hsa-miR-192	21829658
hsa-miR-29a	21829658
hsa-miR-30d	21829658
hsa-miR-320a	21829658
hsa-miR-20b	20651284
hsa-miR-21	20651284
hsa-miR-24	20651284
hsa-miR-15a	20651284
hsa-miR-126	20651284
hsa-miR-191	20651284
hsa-miR-197	20651284
hsa-miR-223	20651284
hsa-miR-320	20651284
hsa-miR-486	20651284
hsa-miR-28-3p	20651284
hsa-miR-133a	20353613
hsa-miR-133b	20353613
hsa-miR-206	20353613
hsa-miR-107	25522185
hsa-miR-195	25138607
hsa-miR-7	24944010
hsa-miR-124a	24944010
hsa-miR-9	24944010
hsa-miR-96	24944010
hsa-miR-15a	24944010
hsa-miR-15b	24944010
hsa-miR-34a	24944010
hsa-miR-195a	24944010
hsa-miR-376	24944010
hsa-miR-107	24944010
hsa-miR-146	24944010

(*Continued*)

miRNA	PMID
hsa-miR-320	24944010
hsa-miR-383	24944010
hsa-miR-181b	24944010
hsa-miR-128a	24944010
hsa-miR-29	24944010
hsa-miR-384-5p	24944010
hsa-miR-126	24944010
hsa-miR-143	24944010
hsa-miR-145	24944010
hsa-miR-33a	24944010
hsa-miR-33b	24944010
hsa-miR-21	24944010
hsa-miR-133a	24944010
hsa-miR-133b	24944010
hsa-miR-223	24944010
hsa-miR-103	24944010
hsa-miR-29a	24944010
hsa-miR-27b	24944010
hsa-miR-15b	25403480
hsa-miR-16	25403480
hsa-miR-17	25403480
hsa-miR-451	25403480
hsa-miR-106b	25403480
hsa-miR-103	25403480
hsa-miR-425	25403480
hsa-miR-106a	25403480
hsa-miR-10b	25403480
hsa-miR-25	25403480
hsa-miR-93	25403480
hsa-miR-363	25403480
hsa-miR-107	25403480
hsa-miR-191	25403480
hsa-miR-30e*	25403480
hsa-let-7i	25403480
hsa-miR-20a	25403480
hsa-miR-223	25403480
hsa-miR-92a	25403480
hsa-miR-20b	25403480

(*Continued*)

miRNA	PMID
hsa-miR-18a	25677225
hsa-miR-221	25677225
hsa-miR-144	25677225
hsa-miR-320a	25677225
hsa-miR-375	25677225
hsa-miR-589	25677225
hsa-miR-665	25677225
hsa-miR-376a	25677225
hsa-miR-140-5p	25677225
hsa-miR-374b	25677225
hsa-miR-155	25677225
hsa-miR-20b	25677225
hsa-miR-126a	25677225
hsa-miR-342-3p	25677225
hsa-miR-107	25677225
hsa-miR-29a	25677225
hsa-miR-34a	25677225
hsa-miR-142-3p	25677225
hsa-miR-106b	25677225
hsa-miR-146a	18633110
hsa-miR-107	19689793
hsa-miR-24-1	20651284
hsa-miR-24-2	20651284
hsa-miR-320a	20651284
hsa-miR-146a	21249428
hsa-miR-143	21441927
hsa-miR-103a-1	21654750
hsa-miR-103a-2	21654750
hsa-miR-144	21829658
hsa-miR-483	22223106
hsa-miR-107	22645244
hsa-miR-126	23144172
hsa-miR-99a	23762265
has-miR-26a	25961460

REFERENCES

1. Park YY, Kim SB, Han HD, Sohn BH, Kim JH, Liang J, Lu Y, Rodriguez-Aguayo C, Lopez-Berestein G, Mills GB, Sood AK, Lee JS. Tat-activating regulatory DNA-binding protein regulates glycolysis in hepatocellular carcinoma by regulating the platelet isoform of phosphofructokinase through microRNA 520. Hepatology. 2013; 58:182–191.
2. Jackson RC, Lui MS, Boritzki TJ, Morris HP, Weber G. Purine and pyrimidine nucleotide patterns of normal, differentiating, and regenerating liver and of hepatomas in rats. Cancer research. 1980; 40:1286–1291.
3. Meng F, Henson R, Wehbe-Janek H, Ghoshal K, Jacob ST, Patel T. MicroRNA-21 regulates expression of the PTEN tumor suppressor gene in human hepatocellular cancer. Gastroenterology. 2007; 133:647–658.
4. Garofalo M, Di Leva G, Romano G, Nuovo G, Suh SS, Ngankeu A, Taccioli C, Pichiorri F, Alder H, Secchiero P, Gasparini P, Gonelli A, Costinean S, Acunzo M, Condorelli G, Croce CM. miR-221&222 regulate TRAIL resistance and enhance tumorigenicity through PTEN and TIMP3 downregulation. Cancer cell. 2009; 16:498–509.
5. Dolce V, Cappello AR, Lappano R, Maggiolini M. Glycerophospholipid synthesis as a novel drug target against cancer. Current molecular pharmacology. 2011; 4:167–175.
6. Ho JC, Cheung ST, Leung KL, Ng IO, Fan ST. Decreased expression of cytochrome P450 2E1 is associated with poor prognosis of hepatocellular carcinoma. International journal of cancer Journal international du cancer. 2004; 111:494–500.
7. Su H, Yang JR, Xu T, Huang J, Xu L, Yuan Y, Zhuang SM. MicroRNA-101, down-regulated in hepatocellular carcinoma, promotes apoptosis and suppresses tumorigenicity. Cancer research. 2009; 69:1135–1142.
8. Leng J, Han C, Demetris AJ, Michalopoulos GK, Wu T. Cyclooxygenase-2 promotes hepatocellular carcinoma cell growth through Akt activation: evidence for Akt inhibition in celecoxib-induced apoptosis. Hepatology. 2003; 38:756–768.
9. Zhou L, Wang Q, Yin P, Xing W, Wu Z, Chen S, Lu X, Zhang Y, Lin X, Xu G. Serum metabolomics reveals the deregulation of fatty acids metabolism in hepatocellular carcinoma and chronic liver diseases. Analytical and bioanalytical chemistry. 2012; 403:203–213.
10. Liang L, Wong CM, Ying Q, Fan DN, Huang S, Ding J, Yao J, Yan M, Li J, Yao M, Ng IO, He X. MicroRNA-125b suppressesed human liver cancer cell proliferation and metastasis by directly targeting oncogene LIN28B2. Hepatology. 2010; 52:1731–1740.
11. Yuan JM, Gao YT, Ong CN, Ross RK, Yu MC. Prediagnostic level of serum retinol in relation to reduced risk of hepatocellular carcinoma. Journal of the National Cancer Institute. 2006; 98:482–490.
12. Newsome PN, Beldon I, Moussa Y, Delahooke TE, Poulopoulos G, Hayes PC, Plevris JN. Low serum retinol levels are associated with hepatocellular carcinoma in patients with chronic liver disease. Alimentary pharmacology & therapeutics. 2000; 14:1295–1301.
13. Ogretmen B, Hannun YA. Biologically active sphingolipids in cancer pathogenesis and treatment. Nature reviews Cancer. 2004; 4:604–616.
14. Silins I, Nordstrand M, Hogberg J, Stenius U. Sphingolipids suppress preneoplastic rat hepatocytes *in vitro* and *in vivo*. Carcinogenesis. 2003; 24:1077–1083.
15. Kandyba AG, Kobliakov VA, Somova OG, Dyatlovitskaya EV. Change in contents of biologically active sphingolipids modulating cell growth and survival in hepatoma 27 compared to rat liver. Biochemistry Biokhimiia. 2004; 69:497–500.
16. Kathagen A, Schulte A, Balcke G, Phillips HS, Martens T, Matschke J, Gunther HS, Soriano R, Modrusan Z, Sandmann T, Kuhl C, Tissier A, Holz M, Krawinkel LA, Glatzel M, Westphal M, et al. Hypoxia and oxygenation induce a metabolic switch between pentose phosphate pathway and glycolysis in glioma stem-like cells. Acta neuropathologica. 2013; 126:763–780.
17. Lei S, Zavala-Flores L, Garcia-Garcia A, Nandakumar R, Huang Y, Madayiputhiya N, Stanton RC, Dodds ED, Powers R, Franco R. Alterations in energy/redox metabolism induced by mitochondrial and environmental toxins: a specific role for glucose-6-phosphate-dehydrogenase and the pentose phosphate pathway in paraquat toxicity. ACS chemical biology. 2014; 9:2032–2048.
18. Granata OM, Carruba G, Montalto G, Miele M, Bellavia V, Modica G, Blomquist CH, Castagnetta LA. Altered androgen metabolism eventually leads hepatocellular carcinoma to an impaired hormone responsiveness. Molecular and cellular endocrinology. 2002; 193:51–58.
19. De Maria N, Manno M, Villa E. Sex hormones and liver cancer. Molecular and cellular endocrinology. 2002; 193:59–63.
20. Christofk HR, Vander Heiden MG, Harris MH, Ramanathan A, Gerszten RE, Wei R, Fleming MD, Schreiber SL, Cantley LC. The M2 splice isoform of pyruvate kinase is important for cancer metabolism and tumour growth. Nature. 2008; 452:230–233.
21. Vander Heiden MG. Targeting cancer metabolism: a therapeutic window opens. Nature reviews Drug discovery. 2011; 10:671–684.
22. Fu L, Dong SS, Xie YW, Tai LS, Chen L, Kong KL, Man K, Xie D, Li Y, Cheng Y, Tao Q, Guan XY. Down-regulation of tyrosine aminotransferase at a frequently deleted region 16q22 contributes to the pathogenesis of hepatocellular carcinoma. Hepatology. 2010; 51:1624–1634.
23. Huang ZZ, Chen C, Zeng Z, Yang H, Oh J, Chen L, Lu SC. Mechanism and significance of increased glutathione level in human hepatocellular carcinoma and liver regeneration.

FASEB journal : official publication of the Federation of American Societies for Experimental Biology. 2001; 15:19–21.
24. Zou Y, Song Y, Yang W, Meng F, Liu H, Zhong Z. Galactose-installed photo-crosslinked pH-sensitive degradable micelles for active targeting chemotherapy of hepatocellular carcinoma in mice. Journal of controlled release: official journal of the Controlled Release Society. 2014; 193:154–161.
25. Sorensen M, Frisch K, Bender D, Keiding S. The potential use of 2-[(1)(8)F]fluoro-2-deoxy-D-galactose as a PET/CT tracer for detection of hepatocellular carcinoma. European journal of nuclear medicine and molecular imaging. 2011; 38:1723–1731.
26. Ai W, Takaishi S, Wang TC, Fleming JV. Regulation of L-histidine decarboxylase and its role in carcinogenesis. Progress in nucleic acid research and molecular biology. 2006; 81:231–270.
27. Murray GI, Taylor MC, Burke MD, Melvin WT. Enhanced expression of cytochrome P450 in stomach cancer. Br J Cancer. 1998; 77:1040–1044.
28. Wideroff L, Vaughan TL, Farin FM, Gammon MD, Risch H, Stanford JL, Chow WH. GST, NAT1, CYP1A1 polymorphisms and risk of esophageal and gastric adenocarcinomas. Cancer Detect Prev. 2007; 31:233–236.
29. Durak ZE, Buber S, Kocaoglu EH, Ozturk B. Static magnetic field inhibits 5′ nucleotidase activity in cancerous and non-cancerous human gastric tissues. Electromagn Biol Med. 2014:1–5.
30. Hu KW, Chen FH, Ge JF, Cao LY, Li H. Retinoid receptors in gastric cancer: expression and influence on prognosis. Asian Pac J Cancer Prev. 2012; 13:1809–1817.
31. Cho LY, Yang JJ, Ko KP, Ma SH, Shin A, Choi BY, Han DS, Song KS, Kim YS, Chang SH, Shin HR, Kang D, Yoo KY, Park SK. Genetic susceptibility factors on genes involved in the steroid hormone biosynthesis pathway and progesterone receptor for gastric cancer risk. PLoS One. 2012; 7(10):e47603.
32. Kominea A, Konstantinopoulos PA, Kapranos N, Vandoros G, Gkermpesi M, Andricopoulos P, Artelaris S, Savva S, Varakis I, Sotiropoulou-Bonikou G, Papavassiliou AG. Androgen receptor (AR) expression is an independent unfavorable prognostic factor in gastric cancer. J Cancer Res Clin Oncol. 2004; 130:253–258.
33. Benjamin DI, Louie SM, Mulvihill MM, Kohnz RA, Li DS, Chan LG, Sorrentino A, Bandyopadhyay S, Cozzo A, Ohiri A, Goga A, Ng SW, Nomura DK. Inositol phosphate recycling regulates glycolytic and lipid metabolism that drives cancer aggressiveness. ACS Chem Biol. 2014; 9:1340–1350.
34. Liu X, Wang X, Zhang J, Lam EK, Shin VY, Cheng AS, Yu J, Chan FK, Sung JJ, Jin HC. Warburg effect revisited: an epigenetic link between glycolysis and gastric carcinogenesis. Oncogene. 2010; 29:442–450.
35. Maehara Y, Kusumoto T, Sakaguchi Y, Kusumoto H, Kido Y, Anai H, Sugimachi K. Pyrimidine nucleotide synthesis is more extensive in poorly differentiated than in well-differentiated human gastric carcinoma. Cancer. 1989; 63:96–101.
36. Meng X, Liu Y, Liu B. Glutathione S-transferase M1 null genotype meta-analysis on gastric cancer risk. Diagn Pathol. 2014; 9:122.
37. Cai L, Yu SZ, Zhang ZF. Glutathione S-transferases M1, T1 genotypes and the risk of gastric cancer: a case-control study. World J Gastroenterol. 2001; 7:506–509.
38. Fan XM, Jiang XH, Gu Q, Ching YP, He H, Xia HH, Lin MC, Chan AO, Yuen MF, Kung HF, Wong BC. Inhibition of Akt/PKB by a COX-2 inhibitor induces apoptosis in gastric cancer cells. Digestion. 2006; 73(2–3):75–83.
39. Wang Z, Chen JQ, Liu JL. COX-2 Inhibitors and Gastric Cancer. Gastroenterol Res Pract. 2014; 2014:132320.
40. Kwon OH, Kang TW, Kim JH, Kim M, Noh SM, Song KS, Yoo HS, Kim WH, Xie Z, Pocalyko D, Kim SY, Kim YS. Pyruvate kinase M2 promotes the growth of gastric cancer cells via regulation of Bcl-xL expression at transcriptional level. Biochem Biophys Res Commun. 2012; 423:38–44.
41. Yoo BC, Ku JL, Hong SH, Shin YK, Park SY, Kim HK, Park JG. Decreased pyruvate kinase M2 activity linked to cisplatin resistance in human gastric carcinoma cell lines. International journal of cancer Journal international du cancer. 2004; 108:532–539.
42. Lu Y, Zhang X, Zhang H, Lan J, Huang G, Varin E, Lincet H, Poulain L, Icard P. Citrate induces apoptotic cell death: a promising way to treat gastric carcinoma? Anticancer Res. 2011; 31:797–805.
43. Kosik J, Gil J, Szmigielski S, Beuth J, Pulverer G. Prevention of hepatic metastases by liver lectin blocking with D-galactose in stomach cancer patients. A prospectively randomized clinical trial. Anticancer Res. 1997; 17:1411–1415.
44. Li PH, Wu JX, Zheng JN, Pei DS. A sphingosine kinase-1 inhibitor, SKI-II, induces growth inhibition and apoptosis in human gastric cancer cells. Asian Pac J Cancer Prev. 2014; 15:10381–10385.
45. Li W, Yu CP, Xia JT, Zhang L, Weng GX, Zheng HQ, Kong QL, Hu LJ, Zeng MS, Zeng YX, Li M, Li J, Song LB. Sphingosine kinase 1 is associated with gastric cancer progression and poor survival of patients. Clin Cancer Res. 2009; 15:1393–1399.
46. Xia JF, Liang QL, Hu P, Wang YM, Li P, Luo GA. Correlations of six related purine metabolites and diabetic nephropathy in Chinese type 2 diabetic patients. Clin Biochem. 2009; 42:215–220.
47. Xia J, Wang Z, Zhang F. Association between Related Purine Metabolites and Diabetic Retinopathy in Type 2 Diabetic Patients. Int J Endocrinol. 2014; 2014:651050.

48. Gastaldelli A, Toschi E, Pettiti M, Frascerra S, Quinones-Galvan A, Sironi AM, Natali A, Ferrannini E. Effect of physiological hyperinsulinemia on gluconeogenesis in non-diabetic subjects and in type 2 diabetic patients. Diabetes. 2001; 50:1807–1812.
49. Lin HV, Accili D. Hormonal regulation of hepatic glucose production in health and disease. Cell Metab. 2011; 14:9–19
50. Xia JF, Hu P, Liang QL, Zou TT, Wang YM, Luo GA. Correlations of creatine and six related pyrimidine metabolites and diabetic nephropathy in Chinese type 2 diabetic patients. Clin Biochem. 2010; 43:957–962.
51. Gupte RS, Floyd BC, Kozicky M, George S, Ungvari ZI, Neito V, Wolin MS, Gupte SA. Synergistic activation of glucose-6-phosphate dehydrogenase and NAD(P)H oxidase by Src kinase elevates superoxide in type 2 diabetic, Zucker fa/fa, rat liver. Free Radic Biol Med. 2009; 47:219–228.
52. Nagle CA, An J, Shiota M, Torres TP, Cline GW, Liu ZX, Wang S, Catlin RL, Shulman GI, Newgard CB, Coleman RA. Hepatic overexpression of glycerol-sn-3-phosphate acyltransferase 1 in rats causes insulin resistance. J Biol Chem. 2007; 282:14807–14815.
53. Cao G, Konrad RJ, Li SD, Hammond C. Glycerolipid acyltransferases in triglyceride metabolism and energy homeostasis-potential as drug targets. Endocr Metab Immune Disord Drug Targets. 2012; 12:197–206.
54. Das UN, Rao AA. Gene expression profile in obesity and type 2 diabetes mellitus. Lipids Health Dis. 2007; 6:35.
55. Kim YK, Lee GS, Jung EM, Hyun SH, Hwang WS, Jeung EB. Generation of fibroblasts overexpressing liver-specific PEPCK in a miniature pig model of human type 2 diabetes mellitus. Mol Med Rep. 2012; 6:45–50.
56. Jeong YW, Lee GS, Kim JJ, Park SW, Ko KH, Kang M, Kim YK, Jung EM, Hyun SH, Shin T, Jeung EB, Hwang WS. Establishment of a canine model of human type 2 diabetes mellitus by overexpressing phosphoenolypyruvate carboxykinase. Int J Mol Med. 2012; 30:321–329.
